# Peroxisome-Mediated Reactive Oxygen Species Signals Modulate Programmed Cell Death in Plants

**DOI:** 10.3390/ijms231710087

**Published:** 2022-09-03

**Authors:** Lichao Huang, Yijing Liu, Xiaqin Wang, Cheng Jiang, Yanqiu Zhao, Mengzhu Lu, Jin Zhang

**Affiliations:** State Key Laboratory of Subtropical Silviculture, College of Forestry and Biotechnology, Zhejiang A&F University, Hangzhou 311300, China

**Keywords:** reactive oxygen species, peroxisome, programmed cell death, hormone

## Abstract

Peroxisomes are a class of simple organelles that play an important role in plant reactive oxygen species (ROS) metabolism. Experimental evidence reveals the involvement of ROS in programmed cell death (PCD) in plants. Plant PCD is crucial for the regulation of plant growth, development and environmental stress resistance. However, it is unclear whether the ROS originated from peroxisomes participated in cellular PCD. Enzymes involved in the peroxisomal ROS metabolic pathways are key mediators to figure out the relationship between peroxisome-derived ROS and PCD. Here, we summarize the peroxisomal ROS generation and scavenging pathways and explain how peroxisome-derived ROS participate in PCD based on recent progress in the functional study of enzymes related to peroxisomal ROS generation or scavenging. We aimed to elucidate the role of the peroxisomal ROS regulatory system in cellular PCD to show its potential in terms of accurate PCD regulation, which contribute to environmental stress resistance.

## 1. Introduction

Peroxisomes are organelles associated with reactive oxygen species (ROS) metabolism, which is involved in a series of ROS generation and scavenging mechanisms. The main types of ROS produced in peroxisomes are superoxide anions (O_2_^.−^) and hydrogen peroxide (H_2_O_2_) [1]. This rank has expanded with the discovery of singlet oxygens (^1^O_2_) in peroxisomes [2]. As a key ROS-related organelle, peroxisome is also involved in processes such as programmed cell death (PCD) [3]. In plants, PCD is defined as any form of cell death involving a series of molecular, biochemical, and cellular changes triggered by programmed developmental processes (dPCD, developmentally induced PCD) or by environmental stresses (ePCD, environmentally induced PCD) [4]. Accurate control of plant PCD is of great significance to the regulation of plant growth, development and environmental stress resistance. dPCD is involved in various plant developmental processes such as xylogenesis, trichome differentiation and leaf senescence, while ePCD is a vital counterbalance during plant response to abiotic and biotic stress. It is well studied that programmed developmental processes or environmental stresses disrupt the balance between ROS production and scavenging, leading to increased cellular ROS levels [5]. The accumulation of cellular ROS acts as a signaling molecule to modulate gene expression or triggers oxidative damages to proteins, DNA and lipids, leading to cell damage and even cell death [4,5]. Therefore, the regulation of ROS level seems to be an effective way to adjust PCD to regulate plant development and environmental stress resistance.

In most of the studies related to ROS-triggered plant PCD, rough information about ROS content variation is available. So, in most cases, we acquire pathways that trigger ROS accumulation to promote plant PCD, but how ROS level changed is easily overlooked. The dearth of information on ROS regulation mechanism is partly attributed to the lack of digging for ROS sources. ROS come from different sources in cells, including peroxisome, mitochondrion, chloroplast, endoplasmic reticulum, cell membrane and cell wall [6,7,8]. Peroxisome has strong reactive oxygen generation and scavenging capacity [9] and does not need to undertake serious tasks such as photosynthesis in chloroplast or energy transformation in mitochondrion. Figure out how peroxisome-derived ROS participated in cellular PCD contribute to plant PCD control. However, more attention has been paid to mitochondrion and chloroplast during plant PCD study, it is unclear whether the ROS originated from peroxisomes participated in PCD. Here, we focus on the relationship between peroxisome-derived ROS and PCD to highlight accurate control of plant PCD. We hope our conclusions can be useful to some actual plant production problems such as increasing gradually abiotic stress caused by global climate change. We also discussed the challenges that need to be solved.

## 2. Peroxisomal ROS Generation Mechanism and PCD

**Figure 1:**
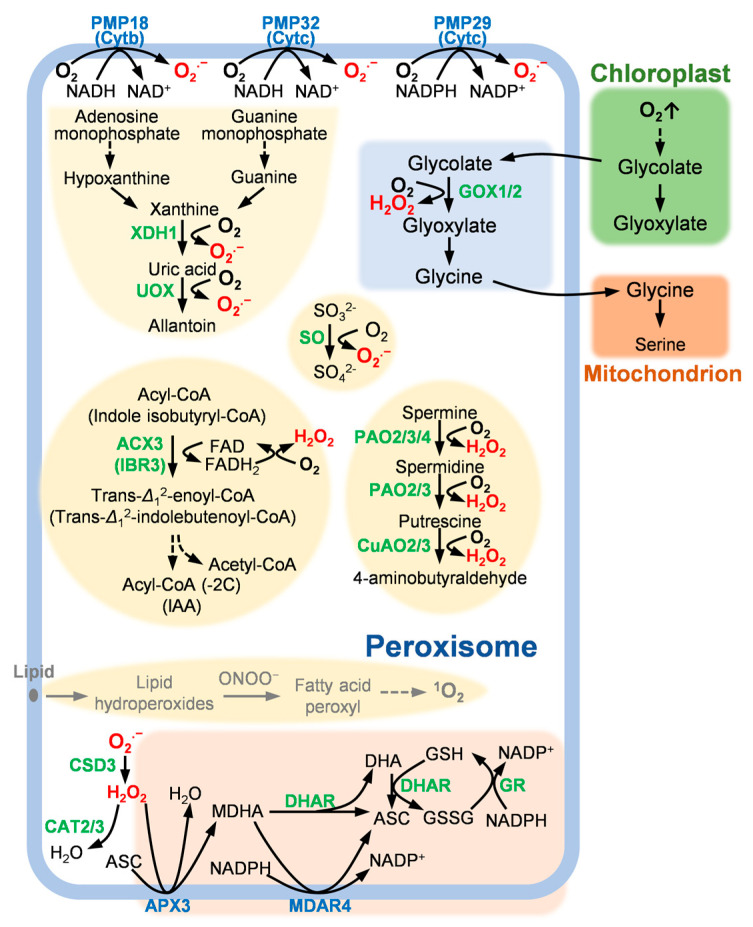
ROS modulation mechanism in peroxisome. PMP, peroxisomal membrane polypeptide; XDH1, xanthine dehydrogenase 1; UOX, urate oxidase; SO, sulfite oxidase; ACX3, acyl-CoA oxidase 3; IBR3, indole-3-butyric acid-response 3; CSD3, copper/zinc superoxide dismutase 3; CAT, catalase; APX3, ascorbate peroxidase 3; MDAR4, monodehydroascorbate reductase 4 (also known as SDP2, sugar-dependent 2); DHAR, dehydroascorbate reductase; GR, glutathione reductase; GOX, glycolate oxidase; PAO, polyamine oxidase; CuAO, copper amino oxidase; NAD^+^, nicotinamide adenine dinucleotide; NADH, reduced form of nicotinamide adenine dinucleotide; NADP^+^, nicotinamide adenine dinucleotide phosphate; NADPH, reduced form of nicotinamide adenine dinucleotide phosphate; FAD^+^, flavin adenine dinucleotide; FADH, reduced form of flavin adenine dinucleotide; IAA, indole-3-aceticacid; MDHA, monodehydroascorbate; ASC, ascorbate; DHA, dehydroascorbate; GSH, glutathione; GSSH, glutathione persulfide; ONOO^−^, peroxynitrite. PMP18, PMP29, PMP32, APX3 and MDAR4 marked in blue letters are peroxisomal membrane proteins.

The mechanism of ROS generation in peroxisome is mainly caused by a series of redox reactions, in which oxygen molecules accept electrons (e^−^) and are converted into different ROS forms (protons also required in some cases) [1,5]. Therefore, the formation of ROS in peroxisomes is related to a series of oxidoreductases and electron carriers. These key enzymes involved in peroxisomal ROS generation mechanism are potential PCD regulators (Figure 1).

### 2.1. The Purine Base Degradation Pathway and PCD

O_2_^.−^ do not participate in plant PCD directly, for they mainly act as a source of H_2_O_2_ [5], which really triggers plant PCD. O_2_^.−^ can be quickly dismutated to H_2_O_2_ by superoxide dismutase (SOD) or react with nitric oxide (NO) to produce peroxynitrite (ONOO^−^), a radial reactive RNS, to induce post-transcriptional modification (described below) [10]. O_2_^.−^ is formed by the acceptance of e^-^ by an oxygen molecule (O_2_). This reaction has been detected in the peroxisomal purine base degradation pathway, which is part of the nucleotide degradation pathway [11]. Xanthine oxidoreductase (XOR) catalyzes the oxidation of xanthine to produce uric acid. During this process, electrons donated by xanthine are transferred to O_2_ through the Fe-S center and the flavin adenine dinucleotide (FAD) of XOR to form O_2_^.−^. In addition, downstream urate oxidase (UOX), which catalyzes the formation of allantoin from uric acid, also catalyzes the formation of O_2_^.−^ [12,13]. XOR contributes more than 30% to oxidative environment in plant, meanwhile, its downstream metabolite allantoin is an antioxidant [14]. Therefore, when considering the purine metabolic pathway as a means of regulating PCD, allantoin as a plant antioxidant should also be taken into consideration. Xanthine dehydrogenase (XDH) is a prominent form of XOR [11]. *Arabidopsis Xanthine Dehydrogenase 1* (*AtXDH1*) dsyfunction mutant exhibits a senescence phenotype with increased ROS level, suggesting that allantoin produced in this pathway as an antioxidant has a stronger effect than the O_2_^.−^ production by AtXDH1 activity [14]. Lower organic nitrogen levels were also detected in *Atxdh1* mutant, as allantoin also acts as a nitrogen source in plant [15]. It seems that enhancing this metabolic pathway has the potential to reduce ROS levels as well as enhance nitrogen levels in plants. In addition, considering that the accumulation of uric acid is harmful to peroxisome [13], UOX level should also be considered in this pathway to avoid uric acid accumulation.

Another source of O_2_^.−^ are the reaction catalyzed by sulfite oxidase [16] and the electron transport chain embedded in peroxisomal membrane [14]. Pea leaf peroxisomal membrane polypeptide PMP18, PMP29 and PMP32 are proposed electron carrier that accepts e^−^ and delivers it to O_2_ to form O_2_^.−^ [17]. However, among studies related to this kind of electron transport chains, more attention has been paid to mitochondrion and chloroplast, the peroxisomal part is still missing. 

### 2.2. Photorespiratory Cycle and PCD

Compared to other types of ROS, H_2_O_2_ owe a longer lifetime, makes it stable enough to pass through the peroxisomal membrane to contribute to cellular ROS level. It has been well summarized that the cellular H_2_O_2_ acts as signaling molecule to regulate ROS-specific transcription factors at low content, while triggers oxidative modification of DNA and proteins at high content, leading to cell damage. [4,5] The main mechanism of H_2_O_2_ production is the photorespiratory pathway, since 70% of the total H_2_O_2_ generated in photosynthetic tissues is mainly catalyzed by glycolate oxidase (GOX) in peroxisomes [18]. The photorespiratory cycle involves chloroplast, peroxisome and mitochondrion. The peroxisomal GOX converts glycolate transferred from chloroplasts into glyoxylate, during which H_2_O_2_ is produced [19]. GOX was involved in cellular PCD regulation. The PCD phenotype of the *CATALASE 2* dysfunction mutant is alleviated by *GOX1* abnormal [20], possibly in part by alleviating cellular ROS pressure. However, a leaf senescence phenotype can be found in *Arabidopsis* by simultaneous inhibiting the expression of *GOX1* and *GOX2*, two major GOXs in the leaf photorespiratory [21]. This indicates that the PCD process caused by transcriptional level alteration of these enzymes cannot be simply attributed to change in ROS, a byproduct of photorespiratory pathways. Although it is energy-costly, serious blocking of photorespiratory cause leaf senescence phenotype as well [21]. However, declined H_2_O_2_ levels and cell-death associated metabolite levels are detected in *Arabidopsis* when only *GOX2* gene is functionally abnormal [19]. So, there is a balance between ROS level variation and photorespiratory cycle maintenance, moderately adjustment of GOX1 or GOX2 may result in ROS level change as well as an appropriate reduction in the energy-costly photorespiratory cycle.

### 2.3. The Fatty Acid β-Oxidation Pathway and PCD

Fatty acid β-oxidation is an important part of lipid catabolism, by which fatty acids are broken down into acetyl-CoA and transferred into mitochondria for glucose metabolism. Acyl-CoA oxidase (ACX) acts as a flavoprotein oxidase to catalyze the first reaction of peroxisomal β-oxidation. Two e^−^ from acyl-CoA are transferred to the cofactor FAD of acyl-CoA oxidase and then to O_2_ to form H_2_O_2_ [22]. Increasing fatty acid flux into peroxisome for the β-oxidation result in ROS accumulation, vice versa [23]. Furthermore, jasmonic acid (JA) is also produced by this pathway. 3-oxo-2-(2′-pentenyl)-cyclopentane-1-octanoic acid (OPC-8:0) go through three cycles of this pathway to form JA [24], which process may be influenced by GOX2 [19]. Acyl-CoA oxidase also catalyzes the peroxisomal IAA metabolic pathways and produces H_2_O_2_, in which indole-3-butyric acid-response 3 (IBR3) acts as an acyl-CoA dehydrogenase/oxidase-like protein and catalyze the conversion of indole-3-butyric acid (IBA) to indole-3-butyric acid (IAA) [25]. The *IBR3* dysfunction has no impact on either fatty acid β-oxidation or JA synthesis, but the IAA synthesis is ACX involved [25]. Among the three products (H_2_O_2_, JA and IAA) of the fatty acid β-oxidation pathway, H_2_O_2_ and IAA trigger peroxisome-induced PCD, while JA exhibits an inhibitory effect [26]. Accumulation of intermediates cause by serious block of lipid catabolism also causes cell death. The *Arabidopsis acx3acx4* double mutant exhibits embryo lethal phenotype due to accumulation of toxic levels of acyl-CoAs [27]. However, ACX3 activity suppression results in a non-fatal plant with reduced IAA and JA synthesis [28]. 

### 2.4. The Polyamine Oxidation Pathway and PCD

Another important pathway related to the production of H_2_O_2_ is the polyamine oxidation pathway. In this pathway, polyamine oxidase (PAO) converts spermine to spermidine or spermidine to putrescine and produces H_2_O_2_ [29]. Putrescine is then converted to 4-aminobutyraldehyde by copper amino oxidase (CuAO), accompanied by H_2_O_2_ production [30]. PAOs located in peroxisome has been proved to participate in polyamine oxidation in *Arabidopsis* and rice (*Oryza sativa*) [29,31,32]. Knock out of *OsPAO5* in rice results in reduced H_2_O_2_ production [32]. The *OsPAO5* expression can be induced by spermidine [32]. That explain why spermidine supply to tobacco plants result in H_2_O_2_ accumulation by enhancing the polyamine oxidation pathway and triggers programmed cell death [33]. However, like the fatty acid β-oxidation pathway, intermediates from polyamine metabolism also play important roles in plant senescence or environmental stress response [29,31]. So, more experimental evidence is needed to connect the polyamine oxidation pathway-derived ROS with PCD.

### 2.5. Other ROS Generation Mechanism

^1^O_2_ signal was newly detected in *Arabidopsis* root peroxisome in dark environment conditions using ^1^O_2_-specific fluorescence probe [2]. ^1^O_2_ is mainly produced in chloroplast for its light-motivated generation pathway [34]. However, Dark condition and the short lifetime of ^1^O_2_ avoid the possibility that the ^1^O_2_ detected in peroxisome may diffuse from chloroplast. This indicates that peroxisome also has a ^1^O_2_ generation pathway. The mechanism of ^1^O_2_ generation in plant peroxisome has not been reported. Miyamoto et al. [35] studied the possibility of producing ^1^O_2_ from hydroperoxides under the condition of metal ions or ONOO^−^. In peroxisome, biological membrane are potential targets of ROS or nitrogen species (RNS) to form lipid hydroperoxides or amino acid hydroperoxides, suggesting that this is a potential ^1^O_2_ generation pathway in peroxisome. It is predicted that ^1^O_2_, which accumulated in chloroplast, controls nuclear gene activities through intermediate components such as lipids or fatty acids, resulting in cell damage [36]. However, more experimental evidence is needed to clarify the relation between peroxisome-derived ^1^O_2_ with PCD. 

## 3. Peroxisomal ROS Scavenging Mechanism and PCD

**Figure 2:**
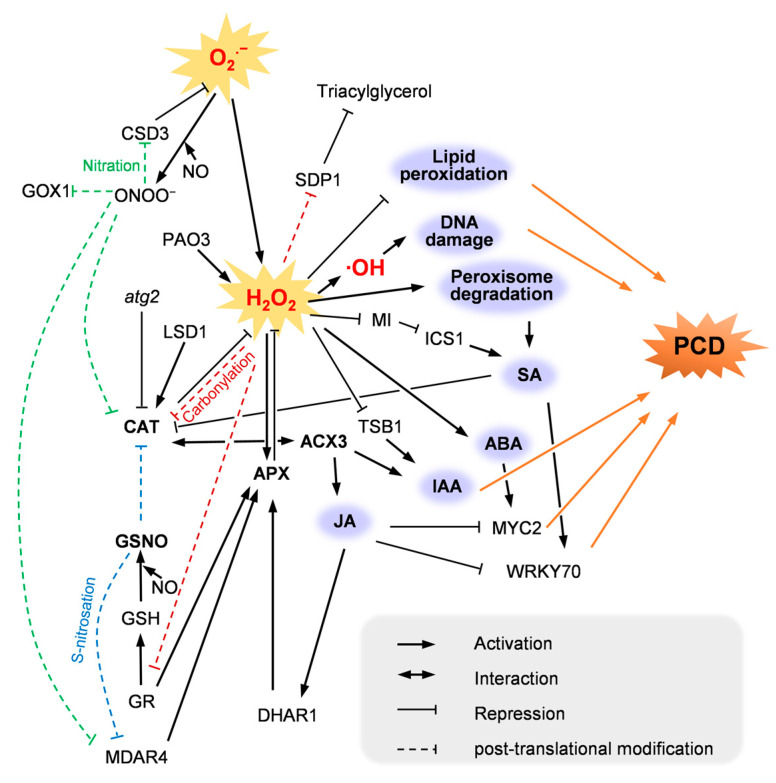
The regulatory mechanism of peroxisome-derived ROS involved in PCD. LSD1, Lesion Simulating Disease 1; ATG2/PEUP1, Autophagy-related 2/Peroxisome Unusual Positioning 1; SDP1, sugar-dependent 1; ICS1, isochorismate synthase 1; TSB1, Tryptophan Synthase Beta-Subunit 1; GSNO, *S*-nitrosoglutathione; NO, nitric oxide; ONOO^−^, peroxynitrite; ·OH, hydroxyl radical; MI, *myo*-inositol; SA, salicylic acid; ABA, abscisic acid; IAA, indole-3-butyric acid; JA, jasmonic acid.

The ROS scavenging mechanism in peroxisome is supported by SOD, catalase (CAT) and ascorbate–glutathione (ASC–GSH) cycle (Figure 1), among which, SOD and CAT catalyze independent reactions. So, compared with peroxisomal ROS generation enzymes, which are involved in different metabolic pathways, ROS scavenging enzymes seems to be more appropriate for peroxisome-derived plant PCD study (Figure 2).

### 3.1. SOD and PCD

SOD is known to catalyze O_2_^.−^ to H_2_O_2_ with its cofactor (metal ion) as an intermediate electron carrier [37]. Therefore, this process also contributes to H_2_O_2_ accumulation. The *Arabidopsis* copper/zinc SOD3 (AtCSD3) has been proved take part in peroxisomal O_2_^.−^ and H_2_O_2_ conversion [38]. Although other two CSD (AtCSD1 located in cytoplasm, AtCSD2 located in chloroplasts) in *Arabidopsis* has been reported involved in H_2_O_2_-mediated cell death [39], the AtCSD3 part of data is still missing. However, the AtCSD3 activity reduced to 65% by the nitration effect caused by ONOO^−^ [40], which produced by the quickly chemical reaction between O_2_^.−^ and NO [41]. The ONOO^−^ production reaction can be enhanced under stress conditions [41]. Further, in *Arabidopsis*, the enzyme activity of GOX1, CAT2, CAT3 and MDAR4 (described below) are also inhibited by the nitration action of ONOO^−^. However, little is known about the balance among O_2_^.−^, H_2_O_2_, ONOO^−^ and peroxisomal SOD, as well as their effect on plant PCD.

### 3.2. CAT and PCD

CAT is the principal H_2_O_2_ scavenging enzyme. Its cofactor heme acts as an intermediate electron carrier in redox reactions to convert H_2_O_2_ to H_2_O [42]. Knockout of the dominant CAT in *Arabidopsis*, peroxisomal *CAT2*, resulted in decreased growth and increased cell death [43]. H_2_O_2_ can be quickly converted to hydroxyl radical (·OH), a toxic molecule that can break DNA hydrogen bonds. Peroxisomal CAT contributes to cellular H_2_O_2_ regulation to protect the plant genomes against H_2_O_2_-induced DNA damage [44,45]. Mass spectrometry-based proteomic analysis revealed that both peroxisomal CAT2 and CAT3 can physically interact with PCD negative regulator Lesion Simulating Disease1 (LSD1) in *Arabidopsis* [3]. The *LSD1* dysfunction mutant showed a similar phenotype to catalase-deficient plants with reduced catalase activity [46], suggesting that LSD1 may suppress PCD by positively regulating CAT2 and CAT3 activity to scavenge ROS. Additionally, the increased cell death phenotype in *lsd1* mutant can be rescued by blocking salicylic acid (SA) accumulation [43]. In consistent, increased peroxisomal H_2_O_2_ trigger isochorismate synthase ICS1 to promote SA synthesis [47]. This pathway is *myo*-inositol (MI) involved [48]. The high oxidative stress in *cat2* represses the production of MI, break the inhibition of MI on ICS1 transcription [43]. In *Arabidopsis Autophagy-related 2* mutant *atg2*, aggregated peroxisomes filled with inactive catalase were detected, which exhibited elevated H_2_O_2_ level and had an SA-dependent early senescence phenotype and spontaneous cell death [49]. Additionally, accumulation of peroxisomes was also detected in *cat2* mutant, implying that ATG2 dysfunction may lead to inactivation of CAT2, thereby inducing H_2_O_2_ to trigger peroxisome aggregation and ultimately SA-induced PCD [49,50]. The experimental evidence suggests that the peroxisome-derived H_2_O_2_ regulation of plant PCD is SA dependent.

In addition to SA, other hormones are involved in peroxisomal catalase-regulated PCD. Kaurilind et al. [26] used 56 *cat2* double/triple mutants to analyze the regulatory mechanism of the plant PCD induced by peroxisomal H_2_O_2_. As a result, either inhibition of the abscisic acid (ABA), IAA or SA signaling pathway moderates the PCD phenotype of *cat2*, while the suppression of the JA synthesis pathway enhanced the PCD phenotype, suggesting that these hormones were involved in peroxisomal H_2_O_2_-triggered PCD. Furthermore, either dysfunction of PCD related transcription factor MYC2 or WRKY70 alleviated the PCD phenotype of *cat2*. It has been proved that the expression of WRKY70 is activated by SA and repressed by JA [51], while the expression of MYC2 is strongly induced by ABA but repressed by JA [52]. The above results indicate that the downstream ABA and SA signaling pathways, triggered by elevated peroxisomal H_2_O_2_ level [26], promote PCD by activating MYC2 and MRKY70 expression, respectively, and the JA signaling pathway may suppress PCD by reducing MYC2 and MRKY70 transcripts.

The feedback regulation of hormones to CAT2 and antagonism action between hormones has also been reported. A feedback expression suppression of JA to *CAT2* is newly found in a MYC2-dependent model [53]. Moreover, SA reduces CAT2 activity, leading to increased H_2_O_2_ levels, which triggers the sulfenylation of Tryptophan Synthase Beta-Subunit 1 (TSB1) to inhibit IAA synthesis [28]. The elevated H_2_O_2_ level suppressed the interaction between CAT2 and ACX3, resulting in a decline in ACX3 activity and reduced IAA and JA synthesis. However, these two hormones are affected to different degrees. IAA level in *cat2* mutants dropped significantly under normal environments, while no observable change was found in JA level [28]. SA also deregulates the physical interaction between peroxisomal GOX and CAT to coordinate H_2_O_2_ levels in rice [54]. Therefore, when trying to regulate PCD through the CAT pathway, hormone level regulation is inevitable.

### 3.3. The ASC–GSH Cycle and PCD

Due to its small size and long lifetime, H_2_O_2_ can easily pass through biomembrane. Membrane-bound peroxisomal ascorbate peroxidase (APX) has a higher affinity for H_2_O_2_ than CAT and is able to degrade H_2_O_2_ that attempts to escape from peroxisome to H_2_O [55]. The stability of this reaction is dependent on the ASC–GSH cycle. Four enzymes, APX, monodehydro-ascorbate reductase (MDAR), dehydroascorbate reductase (DHAR) and glutathione reductase (GR), compose this cycle to ensure that the ascorbate (ASC) consumed by APX activity can be replenished [56,57]. No direct experimental evidence of the involvement of peroxisomal APX in plant PCD has been reported. In *Arabidopsis* and rice, peroxisomal APX expression was elevated during exogenous H_2_O_2_ treatment and decreased during plant senescence [58,59]. Elevating ascorbate levels in *Arabidopsis* stimulates the production of ABA, IAA and JA [60], suggesting a potential participation of peroxisomal APX in PCD. In terms of other ASC–GSH cycle members, H_2_O_2_ from peroxisome that are MDAR4 functionally deficient diffuse to nearby oil body, causing oxidative damage to lipids and suppressing the triacylglycerol lipase activity of sugar-dependent 1 (SDP1) by carbonylation modification, resulting in blocked carbon source and seedling-lethal phenotype [56,61]. However, the phenotype of *sdp1* is less severe when compared with the *MDAR4* dysfunction mutant [62]; the triacylglycerol accumulation in *sdp1* even protects the cell from oxidative stress, implying that the lethal phenotype in the *MDAR4* dysfunction mutant may be contributed to by significant elevated lipid peroxidation induced by H_2_O_2_ released from peroxisome, which has been reported to be cell death related [5]. Alteration of the peroxisomal ASC–GSH cycle is just like installing a release switch to control H_2_O_2_ emission to other parts of the cell.

In addition, GSH from the ASC–GSH cycle can react with NO to form GSNO. This kind of RNS tends to act on the sulfhydryl group of protein to complete *S*-nitrosation action [63]. The enzyme activity of CAT and MDAR can be suppressed by GSNO-induced *S*-nitrosation, leading to more efficient degradation of CAT by peroxisomal proteases [64,65,66], indicating that the ASC–GSH cycle may take part in CAT level regulation.

## 4. Challenges and Future Perspectives

Existing studies have shown that the regulation of ROS metabolism-related enzymes is a general strategy to modulate ROS-induced PCD. However, exploring the function of ROS in peroxisomes is difficult because ROS can freely pass through biomembrane, and other subcellular structures such as chloroplasts and mitochondria are also important sources of ROS. Furthermore, ROS detection methods are mostly limited to histochemical staining, though transmission electron microscopy is a higher-resolution option for visualizing ROS at the subcellular level, and finding ROS receptors remains a challenging task. Therefore, it is not easy to trace the source of ROS during the study of ROS-triggered PCD. In this case, the deficiency can be partially compensated by using mutants of peroxisomal enzymes involved in ROS metabolism. However, experimental evidence mainly came from studies of the model plant *Arabidopsis*. How these enzymes work in other plants such as poplar and rice needs more attention (Table 1) and more information is needed for pathways related to peroxisomal polyamine oxidation and ^1^O_2_ generation.

Inspired by the peroxisomal ROS regulation pathway, Qin et al. [67] immobilized lactate oxidase and CAT into the Fe_3_O_4_ nanoparticle/indocyanine green co-loaded hybrid nanogels to regulate the intracellular ROS level in cancer cells by manipulating the ratio of lactate oxidase and CAT. To establish rice plants with increased photosynthesis efficiency, Shen et al. [68] introduced a GOC bypass into rice chloroplasts by replacing the subcellular location signal of peroxisomal glycolate oxidase, oxalate oxidase and catalase with chloroplastic transit peptide. These ideas encourage a way of modularized assembling of related enzymes to regulate intracellular ROS levels more precisely. In addition, with the rapid development of genome editing technology, the CRISPR/Cas9 system has also become an efficient way to study the function of the ROS metabolism pathway. Moreover, the development of higher-precision multi-omics technology will also provide the possibility to explore the molecular regulation mechanism of ROS-derived PCD.

## Figures and Tables

**Table 1:** Genes involved in *Arabidopsis* peroxisomal ROS metabolism and their homologous genes in poplar and rice.

Gene Name (*Arabidopsis thaliana*)	Gene ID		
	Dicotyledon		Monocotyledon (*Oryza sativa*)
	*Arabidopsis thaliana*	*Populus trichocarpa*	
*ACX3*	AT1G06290 [28]	Potri.019G092600	LOC_Os06g24704 [69]
*APX3*	AT4G35000 [58]	Potri.009G134100	LOC_Os08g43560 [70]
*CAT2*	AT4G35090 [43]	Potri.002G009800	LOC_Os03g03910 [68]
*CAT3*	AT1G20620 [3]	Potri.005G251600	LOC_Os02g02400
*CSD3*	AT5G18100 [38]	Potri.019G035800	LOC_Os07g46990
*CuAO2*	AT1G31710 [30]	Potri.008G151900	LOC_Os07g38440
*CuAO3*	AT2G42490 [30]	Potri.015G082900	LOC_Os04g40040
*DHAR1*	AT1G19570 [60]	Potri.008G049300	LOC_Os05g02530 [71]
*GOX1*	AT3G14420 [20]	Potri.011G112700	LOC_Os07g05820 [54]
*GOX2*	AT3G14415 [19]	Potri.011G112700	LOC_Os07g05820 [54]
*IBR3*	AT3G06810 [25]	Potri.T030600	LOC_Os07g47820
*MDAR4/SDP2*	AT3G27820 [56]	Potri.001G346200	LOC_Os02g47800
*PAO2*	AT2G43020 [29]	Potri.005G207300	LOC_Os04g53190 [72]
*PAO3*	AT3G59050 [29]	Potri.002G055300	LOC_Os04g53190 [72]
*PAO4*	AT1G65840 [29]	Potri.004G075800	LOC_Os04g57560 [32]
*UOX*	AT2G26230 [13]	Potri.010G242600	LOC_Os01g64520
*XDH1*	AT4G34890 [14]	Potri.009G054600	LOC_Os03g31550

## Data Availability

All data are available upon reasonable request.

## References

[1] Corpas F.J., González-Gordo S., Palma J.M. (2020). Plant Peroxisomes: A Factory of Reactive Species. Front. Plant Sci..

[2] Mor A., Koh E., Weiner L., Rosenwasser S., Sibony-Benyamini H., Fluhr R. (2014). Singlet Oxygen Signatures Are Detected Independent of Light or Chloroplasts in Response to Multiple Stresses. Plant Physiol..

[3] Li Y., Chen L., Mu J., Zuo J. (2013). LESION SIMULATING DISEASE1 Interacts with Catalases to Regulate Hypersensitive Cell Death in Arabidopsis. Plant Physiol..

[4] Jiang C., Wang J., Leng H.-N., Wang X., Liu Y., Lu H., Lu M.-Z., Zhang J. (2021). Transcriptional Regulation and Signaling of Developmental Programmed Cell Death in Plants. Front. Plant Sci..

[5] Farooq M.A., Niazi A.K., Akhtar J., Saifullah, Farooq M., Souri Z., Karimi N., Rengel Z. (2019). Acquiring control: The evolution of ROS-Induced oxidative stress and redox signaling pathways in plant stress responses. Plant Physiol. Biochem..

[6] Janků M., Luhová L., Petřivalský M. (2019). On the Origin and Fate of Reactive Oxygen Species in Plant Cell Compartments. Antioxidants.

[7] Suzuki N., Koussevitzky S., Mittler R., Miller G. (2012). ROS and redox signalling in the response of plants to abiotic stress. Plant Cell Environ..

[8] Gross F., Durner J., Gaupels F. (2013). Nitric oxide, antioxidants and prooxidants in plant defence responses. Front. Plant Sci..

[9] Sandalio L.M., Rodríguez-Serrano M., Romero-Puertas M.C., Río L.A. (2013). Role of Peroxisomes as a Source of Reactive Oxygen Species (ROS) Signaling Molecules. Subcell. Biochem..

[10] Costa T.J., Barros P.R., Arce C., Santos J.D., da Silva-Neto J., Egea G., Dantas A.P., Tostes R.C., Jimenez-Altayo F. (2021). The homeostatic role of hydrogen peroxide, superoxide anion and nitric oxide in the vasculature. Free Radic. Biol. Med..

[11] Corpas F.J., Palma J.M., Sandalio L.M., Valderrama R., Barroso J.B., del Río L.A. (2008). Peroxisomal xanthine oxidoreductase: Characterization of the enzyme from pea (*Pisum sativum L*.) leaves. J. Plant Physiol..

[12] Sandalio L.M., Fernandez V.M., Ruperez F.L., del Rio L.A. (1988). Superoxide Free Radicals are Produced in Glyoxysomes. Plant Physiol..

[13] Hauck O.K., Scharnberg J., Escobar N.M., Wanner G., Giavalisco P., Witte C.-P. (2014). Uric Acid Accumulation in an *Arabidopsis* Urate Oxidase Mutant Impairs Seedling Establishment by Blocking Peroxisome Maintenance. Plant Cell.

[14] Soltabayeva A., Bekturova A., Kurmanbayeva A., Oshanova D., Nurbekova Z., Srivastava S., Standing D., Sagi M. (2022). Ureides are accumulated similarly in response to UV-C irradiation and wounding in *Arabidopsis* leaves but are remobilized differently during recovery. J. Exp. Bot..

[15] Soltabayeva A., Srivastava S., Kurmanbayeva A., Bekturova A., Fluhr R., Sagi M. (2018). Early Senescence in Older Leaves of Low Nitrate-Grown Atxdh1 Uncovers a Role for Purine Catabolism in N Supply. Plant Physiol..

[16] Byrne R.S., Hänsch R., Mendel R.R., Hille R. (2009). Oxidative Half-reaction of *Arabidopsis thaliana* Sulfite Oxidase. J. Biol. Chem..

[17] Lopez-Huertas E., Corpas F.J., Sandalio L.M., Del Rio L.A. (1999). Characterization of membrane polypeptides from pea leaf peroxisomes involved in superoxide radical generation. Biochem. J..

[18] Noctor G., Veljovic-Jovanovic S., Driscoll S., Novitskaya L., Foyer C.H. (2002). Drought and oxidative load in the leaves of C3 plants: A predominant role for photorespiration?. Ann. Bot..

[19] Launay A., Jolivet S., Clement G., Zarattini M., Dellero Y., Le Hir R., Jossier M., Hodges M., Expert D., Fagard M. (2022). DspA/E-Triggered Non-Host Resistance against *E. amylovora* Depends on the *Arabidopsis GLYCOLATE OXIDASE 2* Gene. Int. J Mol. Sci..

[20] Kerchev P., Waszczak C., Lewandowska A., Willems P., Shapiguzov A., Li Z., Alseekh S., Mühlenbock P., Hoeberichts F.A., Huang J. (2016). Lack of GLYCOLATE OXIDASE1, but Not GLYCOLATE OXIDASE2, Attenuates the Photorespiratory Phenotype of CATALASE2-Deficient *Arabidopsis*. Plant Physiol..

[21] Dellero Y., Jossier M., Glab N., Oury C., Tcherkez G., Hodges M. (2016). Decreased glycolate oxidase activity leads to altered carbon allocation and leaf senescence after a transfer from high CO_2_ to ambient air in *Arabidopsis thaliana*. J. Exp. Bot..

[22] Baker A., Graham I.A., Holdsworth M., Smith S.M., Theodoulou F.L. (2006). Chewing the fat: β-oxidation in signalling and development. Trends Plant Sci..

[23] Yu L., Fan J., Xu C. (2019). Peroxisomal fatty acid beta-oxidation negatively impacts plant survival under salt stress. Plant Signal. Behav..

[24] Koo A.J., Chung H.S., Kobayashi Y., Howe G.A. (2006). Identification of a peroxisomal acyl-activating enzyme involved in the biosynthesis of jasmonic acid in *Arabidopsis*. J. Biol. Chem..

[25] Zolman B.K., Nyberg M., Bartel B. (2007). IBR3, a novel peroxisomal acyl-CoA dehydrogenase-like protein required for indole-3-butyric acid response. Plant Mol. Biol..

[26] Kaurilind E., Xu E., Brosche M. (2015). A genetic framework for H_2_O_2_ induced cell death in *Arabidopsis thaliana*. BMC Genom..

[27] Khan B.R., Adham A.R., Zolman B.K. (2011). Peroxisomal Acyl-CoA oxidase 4 activity differs between *Arabidopsis* accessions. Plant Mol. Biol..

[28] Yuan H.M., Liu W.C., Lu Y.T. (2017). CATALASE2 Coordinates SA-Mediated Repression of Both Auxin Accumulation and JA Biosynthesis in Plant Defenses. Cell Host Microbe.

[29] Fincato P., Moschou P.N., Spedaletti V., Tavazza R., Angelini R., Federico R., Roubelakis-Angelakis K.A., Tavladoraki P. (2011). Functional diversity inside the *Arabidopsis* polyamine oxidase gene family. J. Exp. Bot..

[30] Planas-Portell J., Gallart M., Tiburcio A.F., Altabella T. (2013). Copper-containing amine oxidases contribute to terminal polyamine oxidation in peroxisomes and apoplast of *Arabidopsis thaliana*. BMC Plant Biol..

[31] Ono Y., Kim D.W., Watanabe K., Sasaki A., Niitsu M., Berberich T., Kusano T., Takahashi Y. (2012). Constitutively and highly expressed *Oryza sativa* polyamine oxidases localize in peroxisomes and catalyze polyamine back conversion. Amino Acids.

[32] Lv Y., Shao G., Jiao G., Sheng Z., Xie L., Hu S., Tang S., Wei X., Hu P. (2021). Targeted mutagenesis of POLYAMINE OXIDASE 5 that negatively regulates mesocotyl elongation enables the generation of direct-seeding rice with improved grain yield. Mol. Plant.

[33] Tisi A., Federico R., Moreno S., Lucretti S., Moschou P.N., Roubelakis-Angelakis K.A., Angelini R., Cona A. (2011). Perturbation of Polyamine Catabolism Can Strongly Affect Root Development and Xylem Differentiation. Plant Physiol..

[34] Jiang H., Chen Y., Li M., Xu X., Wu G. (2011). Overexpression of SGR results in oxidative stress and lesion-mimic cell death in rice seedlings. J. Integr. Plant Biol..

[35] Miyamoto S., Ronsein G.E., Prado F.M., Uemi M., Correa T.C., Toma I.N., Bertolucci A., Oliveira M.C., Motta F.D., Medeiros M.H. (2007). Biological hydroperoxides and singlet molecular oxygen generation. IUBMB Life.

[36] op den Camp R.G., Przybyla D., Ochsenbein C., Laloi C., Kim C., Danon A., Wagner D., Hideg E., Gobel C., Feussner I. (2003). Rapid induction of distinct stress responses after the release of singlet oxygen in *Arabidopsis*. Plant Cell.

[37] Prakash Sanyal R., Samant A., Prashar V., Sharan Misra H., Saini A. (2018). Biochemical and functional characterization of OsCSD3, a novel CuZn superoxide dismutase from rice. Biochem. J..

[38] Huang C.H., Kuo W.Y., Weiss C., Jinn T.L. (2012). Copper chaperone-dependent and -independent activation of three copper-zinc superoxide dismutase homologs localized in different cellular compartments in *Arabidopsis*. Plant Physiol..

[39] Xing Y., Cao Q., Zhang Q., Qin L., Jia W., Zhang J. (2013). MKK5 regulates high light-induced gene expression of Cu/Zn superoxide dismutase 1 and 2 in *Arabidopsis*. Plant Cell Physiol..

[40] Holzmeister C., Gaupels F., Geerlof A., Sarioglu H., Sattler M., Durner J., Lindermayr C. (2015). Differential inhibition of Arabidopsis superoxide dismutases by peroxynitrite-mediated tyrosine nitration. J. Exp. Bot..

[41] Corpas F.J., del Río L.A., Palma J.M. (2019). Plant peroxisomes at the crossroad of NO and H_2_O_2_ metabolism. J. Integr. Plant Biol..

[42] Mhamdi A., Noctor G., Baker A. (2012). Plant catalases: Peroxisomal redox guardians. Arch. Biochem. Biophys..

[43] Chaouch S., Noctor G. (2010). Myo-inositol abolishes salicylic acid-dependent cell death and pathogen defence responses triggered by peroxisomal hydrogen peroxide. New Phytol..

[44] Vanderauwera S., Suzuki N., Miller G., van de Cotte B., Morsa S., Ravanat J.L., Hegie A., Triantaphylides C., Shulaev V., Van Montagu M.C. (2011). Extranuclear protection of chromosomal DNA from oxidative stress. Proc. Natl. Acad. Sci. USA.

[45] Tyutereva E.V., Dobryakova K.S., Schiermeyer A., Shishova M.F., Pawlowski K., Demidchik V., Reumann S., Voitsekhovskaja O.V. (2018). The levels of peroxisomal catalase protein and activity modulate the onset of cell death in tobacco BY-2 cells via reactive oxygen species levels and autophagy. Funct. Plant Biol..

[46] Mateo A., Muhlenbock P., Rusterucci C., Chang C.C., Miszalski Z., Karpinska B., Parker J.E., Mullineaux P.M., Karpinski S. (2004). LESION SIMULATING DISEASE 1 is required for acclimation to conditions that promote excess excitation energy. Plant Physiol..

[47] Han Y., Chaouch S., Mhamdi A., Queval G., Zechmann B., Noctor G. (2013). Functional analysis of *Arabidopsis* mutants points to novel roles for glutathione in coupling H_2_O_2_ to activation of salicylic acid accumulation and signaling. Antioxid. Redox Sign..

[48] Hu L., Zhou K., Ren G., Yang S., Liu Y., Zhang Z., Li Y., Gong X., Ma F. (2020). *Myo*-inositol mediates reactive oxygen species-induced programmed cell death via salicylic acid-dependent and ethylene-dependent pathways in apple. Hortic. Res..

[49] Kang S., Shin K.D., Kim J.H., Chung T. (2018). Autophagy-related (ATG) 11, ATG9 and the phosphatidylinositol 3-kinase control ATG2-mediated formation of autophagosomes in *Arabidopsis*. Plant Cell Rep..

[50] Wang Y., Nishimura M.T., Zhao T., Tang D. (2011). ATG2, an autophagy-related protein, negatively affects powdery mildew resistance and mildew-induced cell death in *Arabidopsis*. Plant J..

[51] Li J., Brader G., Palva E.T. (2004). The WRKY70 transcription factor: A node of convergence for jasmonate-mediated and salicylate-mediated signals in plant defense. Plant Cell.

[52] Kazan K., Manners J.M. (2013). MYC2: The master in action. Mol. Plant.

[53] Song R.-F., Li T.-T., Liu W.-C. (2021). Jasmonic Acid Impairs *Arabidopsis* Seedling Salt Stress Tolerance Through MYC2-Mediated Repression of *CAT2* Expression. Front. Plant Sci..

[54] Zhang Z., Xu Y., Xie Z., Li X., He Z.H., Peng X.X. (2016). Association-Dissociation of Glycolate Oxidase with Catalase in Rice: A Potential Switch to Modulate Intracellular H_2_O_2_ Levels. Mol. Plant.

[55] Narendra S. (2006). The *Arabidopsis* ascorbate peroxidase 3 is a peroxisomal membrane-bound antioxidant enzyme and is dispensable for *Arabidopsis* growth and development. J. Exp. Bot..

[56] Eastmond P.J. (2007). MONODEHYROASCORBATE REDUCTASE4 Is Required for Seed Storage Oil Hydrolysis and Postgerminative Growth in *Arabidopsis*. Plant Cell.

[57] Foyer C.H., Noctor G. (2011). Ascorbate and Glutathione: The Heart of the Redox Hub. Plant Physiol..

[58] Panchuk I.I., Zentgraf U., Volkov R.A. (2005). Expression of the APX gene family during leaf senescence of *Arabidopsis thaliana*. Planta.

[59] Rosa S.B., Caverzan A., Teixeira F.K., Lazzarotto F., Silveira J.A.G., Ferreira-Silva S.L., Abreu-Neto J., Margis R., Margis-Pinheiro M. (2010). Cytosolic APX knockdown indicates an ambiguous redox responses in rice. Phytochemistry.

[60] Bulley S.M., Cooney J.M., Laing W. (2021). Elevating Ascorbate in *Arabidopsis* Stimulates the Production of Abscisic Acid, Phaseic Acid, and to a Lesser Extent Auxin (IAA) and Jasmonates, Resulting in Increased Expression of *DHAR1* and Multiple Transcription Factors Associated with Abiotic Stress Tolerance. Int. J. Mol. Sci..

[61] Theodoulou F.L., Eastmond P.J. (2012). Seed storage oil catabolism: A story of give and take. Curr. Opin. Plant Biol..

[62] Fan J., Yan C., Roston R., Shanklin J., Xu C. (2014). Arabidopsis lipins, PDAT1 acyltransferase, and SDP1 triacylglycerol lipase synergistically direct fatty acids toward beta-oxidation, thereby maintaining membrane lipid homeostasis. Plant Cell.

[63] Wink D.A., Hanbauer I., Grisham M.B., Laval F., Nims R.W., Laval J., Cook J., Pacelli R., Liebmann J., Krishna M. (1996). Chemical biology of nitric oxide: Regulation and protective and toxic mechanisms. Curr. Top. Cell Regul..

[64] Lozano-Juste J., Colom-Moreno R., Leon J. (2011). *In vivo* protein tyrosine nitration in *Arabidopsis thaliana*. J. Exp. Bot..

[65] Begara-Morales J.C., Sánchez-Calvo B., Chaki M., Mata-Pérez C., Valderrama R., Padilla M.N., López-Jaramillo J., Luque F., Corpas F.J., Barroso J.B. (2015). Differential molecular response of monodehydroascorbate reductase and glutathione reductase by nitration and S-nitrosylation. J. Exp. Bot..

[66] Rodríguez-Ruiz M., González-Gordo S., Cañas A., Campos M.J., Paradela A., Corpas F.J., Palma J.M. (2019). Sweet Pepper (*Capsicum annuum* L.) Fruits Contain an Atypical Peroxisomal Catalase That Is Modulated by Reactive Oxygen and Nitrogen Species. Antioxidants.

[67] Qin X., Wu C., Niu D., Qin L., Wang X., Wang Q., Li Y. (2021). Peroxisome inspired hybrid enzyme nanogels for chemodynamic and photodynamic therapy. Nat. Commun..

[68] Shen B.R., Wang L.M., Lin X.L., Yao Z., Xu H.W., Zhu C.H., Teng H.Y., Cui L.L., Liu E.E., Zhang J.J. (2019). Engineering a New Chloroplastic Photorespiratory Bypass to Increase Photosynthetic Efficiency and Productivity in Rice. Mol. Plant.

[69] Kim M.C., Kim T.H., Park J.H., Moon B.Y., Lee C.H., Cho S.H. (2007). Expression of rice acyl-CoA oxidase isoenzymes in response to wounding. J. Plant Physiol..

[70] Chou T.S., Chao Y.Y., Kao C.H. (2012). Involvement of hydrogen peroxide in heat shock- and cadmium-induced expression of ascorbate peroxidase and glutathione reductase in leaves of rice seedlings. J. Plant Physiol..

[71] Kim Y.S., Kim I.S., Bae M.J., Choe Y.H., Kim Y.H., Park H.M., Kang H.G., Yoon H.S. (2013). Homologous expression of cytosolic dehydroascorbate reductase increases grain yield and biomass under paddy field conditions in transgenic rice (*Oryza sativa* L. *japonica*). Planta.

[72] Liu T., Kim D.W., Niitsu M., Berberich T., Kusano T. (2014). *Oryza sativa* polyamine oxidase 1 back-converts tetraamines, spermine and thermospermine, to spermidine. Plant Cell Rep..

